# Correcting for multiple-testing in multi-arm trials: is it necessary and is it done?

**DOI:** 10.1186/1745-6215-15-364

**Published:** 2014-09-17

**Authors:** James M S Wason, Lynne Stecher, Adrian P Mander

**Affiliations:** MRC Biostatistics Unit Hub for Trials Methodology Research, Institute of Public Health, Robinson Way, Cambridge, CB2 0SR UK; Else-Kröner-Fresenius Center for Nutritional Medicine, Technische Universität München, Gregor-Mendel-Strasse 2, 85350 Freising-Weihenstephan, Munich, Germany

**Keywords:** Family-wise error rate, Multi-arm clinical trial, Multiple-test correction, Type-I error rate

## Abstract

**Background:**

Multi-arm trials enable the evaluation of multiple treatments within a single trial. They provide a way of substantially increasing the efficiency of the clinical development process. However, since multi-arm trials test multiple hypotheses, some regulators require that a statistical correction be made to control the chance of making a type-1 error (false-positive). Several conflicting viewpoints are expressed in the literature regarding the circumstances in which a multiple-testing correction should be used. In this article we discuss these conflicting viewpoints and review the frequency with which correction methods are currently used in practice.

**Methods:**

We identified all multi-arm clinical trials published in 2012 by four major medical journals. Summary data on several aspects of the trial design were extracted, including whether the trial was exploratory or confirmatory, whether a multiple-testing correction was applied and, if one was used, what type it was.

**Results:**

We found that almost half (49%) of published multi-arm trials report using a multiple-testing correction. The percentage that corrected was higher for trials in which the experimental arms included multiple doses or regimens of the same treatments (67%). The percentage that corrected was higher in exploratory than confirmatory trials, although this is explained by a greater proportion of exploratory trials testing multiple doses and regimens of the same treatment.

**Conclusions:**

A sizeable proportion of published multi-arm trials do not correct for multiple-testing. Clearer guidance about whether multiple-testing correction is needed for multi-arm trials that test separate treatments against a common control group is required.

**Electronic supplementary material:**

The online version of this article (doi:10.1186/1745-6215-15-364) contains supplementary material, which is available to authorized users.

## Background

For most diseases there are multiple new treatments at the same stage of clinical development. For example, in oncology there are over 1,500 treatments in the clinical pipeline [1]. With limited resources and patients available, alternative trial designs are needed to maximise the number of treatments tested. Multi-arm designs are an important example of an alternative trial design that substantially improves efficiency over the traditional two-arm randomised controlled trial (RCT).

**Figure 1:**
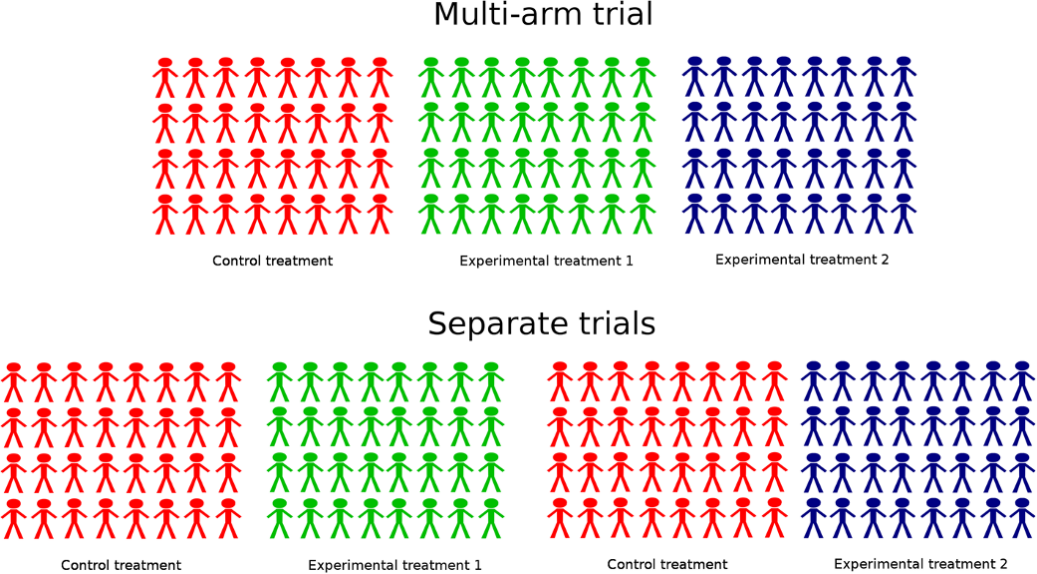
**Illustration of a multi-arm trial and the benefit of a shared control group.** Separate controlled trials will require a greater number of control patients compared to a multi-arm trial with shared control group.

Multi-arm trials vary considerably in design and objective, but have in common that more than two treatment arms are included in the same trial protocol. They evaluate multiple research questions that would otherwise require several trials, and have two main advantages in comparison to separate trials: 1) a reduction in administrative burden; 2) improved efficiency by using shared information. The improved efficiency can be used to reduce the sample size required for a given power, or to maintain the sample size whilst increasing the power to show that one of the experimental treatments is better than control [2]. A common multi-arm design that provides increased efficiency is one that tests multiple experimental arms against a shared control arm. The shared control arm is used for testing the effect of each experimental treatment, reducing the total number of patients needed (see Figure 1). Some other multi-arm trial designs also have this advantage, for example when a single experimental treatment is compared to placebo and an active control [3]. A recent review by Baron *et al*. [4] found that 17.6% of published randomised controlled trials in 2009 were multi-arm.

Despite their common use, there is no consensus in the literature about whether a trial with multiple arms should make a statistical correction for the fact that multiple primary hypotheses are tested in the analysis. In this paper we provide a summary of different viewpoints on the subject, and conduct a literature review to investigate how often recently published multi-arm trials in major medical journals include an adjustment for multiple-testing.

### What is multiple-test correction, and is it necessary for multi-arm trials?

A multi-arm trial has multiple null-hypotheses, each representing a different primary research question. This creates an additional layer of complexity over a trial with one primary null-hypothesis, such as a two-arm RCT. When there is a single null-hypothesis, the significance level (or type-I error rate) is the probability of rejecting the null-hypothesis when it is true. In a multi-arm trial, there are more potential ways in which a false-positive finding can be made: any true null-hypothesis that is rejected will mean that the trial makes a false-positive finding. For example, if four independent true null-hypotheses are tested at 5% significance level the total chance of a false-positive is 19%.

A multiple-testing procedure is a statistical method of adjusting the significance level used for testing each hypothesis so that the chance of making a type-I error is controlled. There are various characteristics that the testing procedure can have. Amongst the strictest is *strong control of the family-wise error rate* (FWER). The FWER is the probability of making at least one type-I error and strong control means that the maximum possible FWER is controlled at a pre-defined level. For example, testing four null-hypotheses would strongly control the FWER at level 0.05 if the maximum possible chance of rejecting a true null-hypothesis is less than or equal to 0.05. *Weak control* of the FWER is similar, but only controls the maximum possible FWER under the subset of situations when all null-hypotheses are true, known as the global null-hypothesis. A procedure that controls the FWER strongly will control it weakly, but not necessarily *vice versa*. Another commonly considered quantity is the false discovery rate (FDR), which is the expected proportion of true null-hypotheses that are rejected. A procedure controlling the FDR would permit true null-hypotheses to be rejected, as long as the expected proportion of true null-hypotheses that are rejected is below a target level; a procedure controlling the FWER would control the probability of rejecting at least one true null-hypothesis. A procedure that controls the FWER will also control the FDR at the same level.

Multiple-testing arises in many areas of biology, not just in clinical trials. For example, many advances in multiple-testing procedure methodology have been motivated by genomics [5] where studies routinely test many thousands of hypotheses in a single study. Some authors, example Rothman [6], claim that multiple-test corrections should never be used in scientific experiments. Rothman argues that advocating multiple-testing adjustment assumes that all null-hypotheses are true, and when that is not the case, it will reduce the power to find genuine associations. However, other authors have subsequently argued that multiple-testing correction is necessary in different clinical trial scenarios. An example of a paper that argues against Rothman’s view is Bender and Lange [7], which provides a discussion of multiple-testing in biomedical and epidemiological research and an overview of methods used to correct for multiple-testing.

In clinical trials, multiple primary hypotheses can arise in several ways, not only due to considering more than two arms. For example, clinical trials commonly assess the performance of a new treatment by recording several outcomes. If the treatment would be declared effective if there is a significant difference in any of the outcomes then there is the potential for an increased type-I error rate. Feise [8] provides a balanced consideration over whether a multiple-testing correction is required in a trial using multiple outcomes and recommends the use of composite measures or selecting a single primary outcome measure in order to avoid the problem entirely. If multiple primary outcomes are used in a confirmatory clinical trial, and any significant result would be grounds for licensing the treatment, then regulators are clear that a multiple-testing adjustment is required [9, 10]. Another situation in which a multiple-testing correction is routinely used is in a trial where the same hypothesis is tested at multiple interim analyses. Again, it is fairly well accepted that in this case a multiple-testing correction is required, with an extensive literature on group-sequential designs that control the type-I error rate and power when interim analyses are used (see Jennison and Turnbull [11] for an extensive summary of methods).

For multi-arm trials, the context of the trial influences whether multiple-testing correction is desirable. If the trial is exploratory, and any findings will be tested in further trials, then there is less need for a multiple-testing correction, as any false-positive findings will not change practice. In fact, recent evidence has shown that from an efficiency standpoint, exploratory multi-arm studies should use high significance levels when they are followed by a confirmatory trial [12].

In confirmatory settings, when the multi-arm trial is designed to provide a definitive answer to the hypotheses being tested, there are conflicting views about the necessity of multiple-test correction. Cook and Farewell [13] argue that if the different hypotheses represent distinct research questions (for example, the effect of distinct experimental treatments in comparison to the control treatment), then it is reasonable to not apply a procedure that strongly controls the FWER. In Bender and Lange [7], a section on experiments with multiple treatments argues that it is mandatory to control the FWER when multiple significance tests are used for primary hypotheses in a confirmatory setting. Hughes [14] makes the argument that multiple-testing adjustment is not necessary when several experimental arms are compared to a control group, as that adjustment would not be needed if the treatments were tested in separate trials. This argument differentiates testing independent treatments rather than considering the question of whether any of the treatments are beneficial. However, multi-arm trials are usually reported in a single paper and the treatment effects are often discussed and interpreted relative to each other. Freidlin *et al*. [15] refines the view of Hughes, arguing that a multiple-testing adjustment is necessary when several doses or schedules of the same treatment are tested against a common control, but not when the treatments are distinct and the multi-arm trial is conducted for efficiency reasons. This distinction is made because any rejected null-hypothesis will result in the new treatment being recommended. Proschan and Waclawiw [16] provide consideration of many sources of multiplicity in clinical trials, including multiple experimental arms. It is stated that multiple-testing adjustment is more necessary when: 1) the hypotheses being tested are more related; 2) the number of comparisons is higher; 3) the degree of controversy is higher (that is whether the trial is aiming to definitely answer a question that has had conflicting results in the literature); 4) when one party stands to benefit from the multiple-testing (for example, several of the treatments in the trial are produced by a single manufacturer). Wason *et al*. [17] argue that for a multi-arm trial, the FWER should be strongly controlled in confirmatory trials, and reported in exploratory trials. This argument was based on the two main regulatory bodies for pharmaceutical trials currently providing advice suggesting that adjustment is required for definitive trials. The European Medicines Agency (EMEA) guidance on multiplicity [10] states that any confirmatory trial with multiple primary null-hypotheses should control the maximum probability of making a type-I error. The Food and Drugs Administration (FDA) (draft) guidance on adaptive designs [18] states that the total study-wise error rate should be controlled in all confirmatory trials, although does not explicitly mention multi-arm trials. To our knowledge, there are no official guidelines on this issue for non-pharmaceutical trials.

Thus there is no unanimous view on the issue of multiple-testing corrections in confirmatory multi-arm trials. There are indications that it is a regulatory requirement, but this would only be relevant for trials that aim to gather evidence to support registration of a drug. There is little evidence about whether correction is done in practice. Baron *et al*. [4] found that around 40% of multi-arm trials published in 2009 adjusted for multiple-testing, although did not distinguish between exploratory and confirmatory trials.

In the next section we investigate what proportion of recently published multi-arm clinical trials corrected for multiple-testing.

## Methods

A literature review of multi-arm trials reported between January 2012 and December 2012 in four major medical journals (*British Medical Journal* (*BMJ*), *The Lancet*, *New England Journal of Medicine* (*NEJM)*, *PLoS Medicine*) was performed. Through searching the electronic content of these journals, potentially eligible articles were identified by one author (LC) based on title and abstract. Full reports of these articles were obtained and assessed for eligibility by all authors independently. To be considered eligible, the article needed to report the main analysis of either an exploratory (Phase 2) or confirmatory (Phase 3) multi-arm trial. Phase 1 studies were excluded. Trials of both parallel group and factorial design were included. Papers where the classification was not clear were discussed between authors until a consensus was reached.

The principal aim of our review was to determine how often the inclusion of more than two arms was taken into account in the design of such trials and summarise the methods used.

## Results

Fifty-nine multi-arm trials, encompassing a wide range of disease areas, were included in our review. Of the total, three were reported in *PLoS Medicine*, nine in the *BMJ*, 18 in *The Lancet* and 29 in *NEJM*. A summary of the characteristics of these trials is given in Table 1.

**Table 1:** **Characteristics of the studies included in the review**

	Exploratory trials (n = 20)	Confirmatory trials (n = 39)	All trials (n = 59)
**Number of study arms**			
3	8 (40%)	22 (56%)	30 (51%)
4	3 (15%)	13 (33%)	16 (27%)
> 4	9 (45%)	4 (10%)	13 (22%)
**Trial design**			
Parallel group	19 (95%)	30 (77%)	49 (83%)
a) Different doses or regimens of same treatment	13	16	29
b) Different treatments	3	8	11
c) Combined treatments	3	6	9
Parallel group with factorial	1 (5%)	9 (23%)	10 (17%)
**Comparisons reported (restricted to parallel group trials)**			
All pairwise	2/19 (11%)	4/30 (13%)	6/49 (12%)
Multiple experimental versus control	11/19 (58%)	19/30 (63%)	30/49 (61%)
Experimental versus multiple controls	1/19 (5%)	2/30 (7%)	3/49 (6%)
Other	5/19 (26%)	5/30 (17%)	10/49 (20%)
***ICD-10*** **Disease classification**			
Certain infectious and parasitic diseases	3 (15%)	5 (13%)	8 (14%)
Neoplasms	0 (0%)	5 (13%)	5 (8%)
Endocrine, nutritional and metabolic diseases	4 (20%)	4 (10%)	8 (14%)
Diseases of the nervous system	1 (5%)	4 (10%)	5 (8%)
Diseases of the circulatory system	5 (25%)	4 (10%)	9 (15%)
Other	7 (35%)	17 (44%)	24 (41%)
**Allocation ratio**			
Equal	18 (90%)	31 (79%)	49 (83%)
Not Equal	2 (10%)	8 (21%)	10 (17%)
**Adjustment procedure**			
None	9 (45%)	21 (54%)	30 (51%)
Bonferroni	2 (10%)	6 (15%)	8 (14%)
Dunnett	0 (0%)	3 (8%)	3 (5%)
Hochberg	1 (5%)	3 (8%)	4 (7%)
Hierarchical (or other closed procedure)	8 (40%)	6 (15%)	14 (24%)
**Sample size calculation reported**			
In report	16 (80%)	30 (77%)	46 (78%)
Referenced or in supplementary material	2 (10%)	6 (15%)	8 (14%)
Not given	2 (10%)	3 (8%)	5 (8%)

*Abbreviation*: *ICD-10*, International Classification of diseases, version 10.

More than half were three-arm trials, with 13 (22%) consisting of more than four arms. The most common trial design was a parallel group comparison of different doses of the same treatment (in addition to control arms). In the majority of multi-arm trials (83%) the allocation ratio was equal between study arms.

Amongst all trials, slightly more than half of the trials did not adjust for multiplicity (51%). Adjustment was slightly higher in trials classified as exploratory (55%) than for trials classed as confirmatory (46%). Of those that did adjust, use of a hierarchical/closed testing approach was most common (24% of included trials), followed by Bonferroni correction (14%). The sample size calculation was not reported/referenced for 5 (8%) studies. We should note that there are many other multiple-testing adjustments available, and the list in Table 1 is not exhaustive.

Table 2 breaks down the frequency of adjustment by the classification of the experimental arms. We note that the total adds up to more than 59 as some trials covered more than one classification (for example, distinct treatments and distinct doses of the same treatment). Amongst trials where the experimental arms included arms with the same treatment at different doses, adjustment was more frequent (62% for exploratory studies, and 67% for confirmatory studies). This was also true for trials where the experimental arms included arms with the same treatment given using different regimens (67% for exploratory and 60% for confirmatory studies). The adjustment was lower amongst trials where the different experimental arms were all separate treatments (50% for exploratory trials and 30% for confirmatory trials). This table also explains why adjustment appears to be higher for exploratory trials than for confirmatory trials - there are a greater number of exploratory trials with different doses or regimens, and these had a higher frequency of adjustment.

**Table 2:** **Frequency of multiple-test correction by nature of experimental arms**

	Exploratory trials	Confirmatory trials
Multiple doses of same treatment	8/13 (62%)	8/12 (67%)
Multiple regimens of same treatment	2/3 (67%)	6/10 (60%)
Separate treatments	3/6 (50%)	6/20 (30%)

Table 3 breaks down the frequency of adjustment by journal. From a logistic regression model that included a factor for journal, there is no significant difference in the frequency of adjustment between journals.

**Table 3:** **Frequency of multiple-test correction by journal**

	Exploratory trials	Confirmatory trials
*BMJ*	0/1 (0%)	2/8 (25%)
*The Lancet*	4/7 (57%)	5/11 (45%)
*NEJM*	7/11 (64%)	10/18 (56%)
*PLoS Medicine*	0/1 (0%)	1/2 (50%)

## Discussion

**Figure 2:**
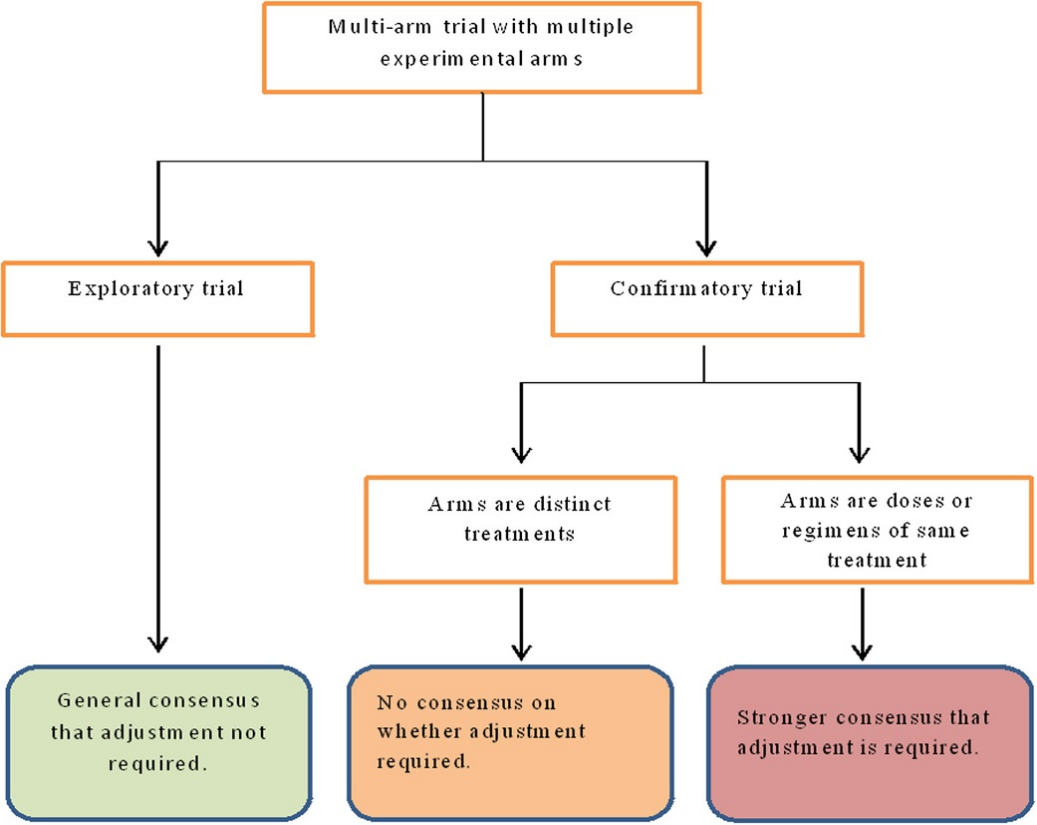
**Flowchart representing consensus from literature on under what circumstances multiple-testing adjustment is necessary.**

Multi-arm trials represent an extremely useful design for improving the efficiency of evaluating novel treatments in a clinical trial. Currently, there are conflicting opinions on whether a multiple-test correction is needed for multi-arm trials. Our opinion is that stringent multiple-testing correction is not required for exploratory trials, and may in fact result in potentially effective treatments being abandoned prematurely. However, it is still relevant to consider the total probability of making a type-I error in exploratory trials, and the overall FWER should be reported. For confirmatory trials, we agree with current guidance expressed by several authors that in trials of several doses or regimens of the same treatment, the FWER should be strongly controlled. In the case where the different arms are separate treatments, the literature is unclear on whether an adjustment is necessary. The current guidance from pharmaceutical regulators appears to suggest that adjustment is necessary, but this is not explicitly stated to be the case for multi-arm trials of separate treatments. Figure 2 represents these recommendations as a flow diagram.

Our literature review provides evidence that multiple-testing correction is not performed in the majority of multi-arm trials, although the adjustment frequency is higher in trials testing several doses or regimens of the same treatment.

We have not considered the various methods that exist for correcting for multiple-testing in this paper, as other thorough overviews of various methods exist; for example, see Bender and Lange [7]. In Additional file 1, some discussion and R code is provided for some available methods. In addition to hypothesis testing, it is often of interest to estimate treatment effects and provide a measure of uncertainty, for example a 95% confidence interval (CI). There is generally a close relationship between hypothesis testing and CI - if a 95% CI does not include a treatment difference of zero, then the null-hypothesis of no difference can be rejected with 5% type-I error rate. The question of multiple-testing correction can also be posed for CIs - should the 95% coverage typically used for CIs be adjusted when several hypotheses are tested? Obviously, if CIs are directly used to test hypotheses in a confirmatory trial, then the same arguments regarding adjustment of *P*-values apply. However, it is less clear that the coverage of a CI requires adjustment if it is only used as the summary of the uncertainty in a treatment’s effect. In that case, we do not see a need to adjust the CI coverage in situations where there is a need to correct the significance level. It is clearer, from their name, that if several 95% CIs are reported, the chance of one of them not containing the true treatment effect will increase. If adjustment of CIs is required, then Bender and Lange [7] discuss some methods that allow this.

## Conclusions

Multiple-testing correction is not performed in a sizable proportion of multi-arm trials published in top-quality medical journals. Confirmatory trials with several doses or regimens of the same treatments should be required to report the overall probability of the trial making a type-I error and apply a multiple-testing correction that controls the FWER at a specified level. More guidance is needed from regulatory agencies about confirmatory trials of several separate treatments.

## Electronic supplementary material

Additional file 1: **Supplementary material describing some multiple-testing procedures in more detail with R code provided.** (DOC 74 KB)

## Abbreviations

*BMJ*: *British Medical Journal*
CI: confidence interval
FDA: Food and Drugs Administration
FDR: false discovery rate
FWER: family-wise error rate
*NEJM*: *New England Journal of Medicine*
RCT: randomised controlled trial.
