# Supplementary material for: Correcting for multiple-testing in multi-arm trials: is it necessary and is it done?
Source: Trials. 2014 Sep 17;15:364. doi: 10.1186/1745-6215-15-364 (PMC4177585; doi:10.1186/1745-6215-15-364)
Supplement: Supplementary file 1 — Additional file 1: Supplementary material describing some multiple-testing procedures in more detail with R code provided. (DOC 74 KB) [file 13063_2014_2233_MOESM1_ESM.doc]

**Supplementary material for “Correcting for multiple-testing in multi-arm trials: is it necessary and is it done?”**

**Description of multiple-testing procedures and R code**

We have not considered the various methods that exist for correcting for multiple testing in the main paper, as other thorough overviews of various methods exist. For example, see Bender and Lange [1]. In this supplementary material, we provide some recommendations on what procedures are used and some technical description of a small subset of commonly used procedures.

In the case of a multi-arm trial testing several experimental arms against a common control, we would recommend that methods that take the resulting correlation between test statistics into account are used, such as Dunnett’s procedure [2] or Dunnett’s step-down procedure [3]. These procedures provide strong control of the FWER which is ideal for confirmatory settings. In the case where all experimental arms are expected to have an effect, or none are (such as when the arms represent different doses of the same treatment, or different schedules), then a two-stage procedure where an omnibus test of the hypothesis that all experimental arms have the same effect as the control arm is followed by individual tests of each arm can be applied. Several two-stage procedures exist and are discussed in Bender and Lange [1]. Their main disadvantage is the second stage only occurs if the omnibus test is positive; the procedure can therefore have low power if not all the experimental arms are better than control. In the case where there are multiple hypotheses but no clear relation between them, then methods such as Bonferroni (see for example [4]), or Holm’s step-down procedure [5] provide strong control of FWER, but will overcorrect when there is in fact correlation between test statistics. We next provide more detailed descriptions of some multiple testing procedures.

*Bonferroni adjustment*

If there are K hypotheses being tested, and the desired overall family-wise error rate (FWER) is $\alpha$, then the Bonferroni adjusted p-value is simply $\alpha/K$. This will typically result in a FWER of below $\alpha$, especially if there is a correlation between the test-statistics used to test the different hypotheses.

R-code:

bonferroni=function(alpha,K)

{

return(rep(alpha/K,K))

}

#example:

> bonferroni(0.05,4)

[1] 0.0125 0.0125 0.0125 0.0125

*Holm’s step-down procedure*

Holm uses Bonferroni adjustments, but in a sequential way. If there are K null ypotheses, each with a p-value derived from a test-statistic, then Holm’s procedure works as follows:

1. order the p-values so that they go from lowest to highest;
2. compare the lowest p-value to $\alpha/K$, and reject the null hypothesis associated with that p-value if the p-value is below;
3. If the null hypothesis tested in step 2 was rejected, compare the second lowest p-value to $\alpha/(K-1)$, and reject the null hypothesis associated with that p-value if the p-value is below;
4. repeat step 3 for the third lowest, fourth lowest p-values etc, comparing the p-value of the jth lowest pvalue to $\alpha/(K-j+1)$ until a null hypothesis is not rejected.

R-code (returns the p-value thresholds in order):

holm=function(alpha,K)

{

return(alpha/(K:1))

}

#example:

> holm(0.05,4)

[1] 0.01250000 0.01666667 0.02500000 0.05000000

*Dunnett*

Dunnett takes the correlation between test statistics into account when deriving the stopping boundaries. We assume that the test statistics are asymptotically normally distributed, as would be the case with normally distributed, binary or time-to-event endpoints. We further assume that the test statistics are standardised, so that they have variance 1 under the null hypothesis and that the allocation to each treatment is equal. The maximum FWER will be obtained when all experimental treatments have the same effect as the control treatment. The joint distribution of the test statistics in this case will be asymptotically normal with mean vector 0, and covariance matrix $\Sigma$, where the diagonal entries of $\Sigma$ are 1, and the (i,j)th non-diagonal entry is 0.5.

If a critical value, c, is used to declare significance, the probability of not rejecting any null hypothesis is:

$$\int_{-\infty}^{c} \int_{-\infty}^{c} \ldots\int_{-\infty}^{c} f((x_{1},x_{2},\ldots,x_{K}),0,\Sigma)dx_{K}dx_{K-1}\ldots dx_{1}$$

where $f(x,\mu,\Sigma)$ is the probability density function of a multivariate normal distribution with mean $\mu$ and covariance matrix $\Sigma$, evaluated at vector x. A critical value is then chosen such that equation is equal to $1-\alpha$. The p-value threshold is equal to

$$1-\Phi(c)$$

where $\Phi()$ is the cumulative density function of the standard (univariate) normal distribution.

R-code:

library(mvtnorm)

#fwer_dunnett returns the difference between the FWER for a given critical value and the target FWER

fwer_dunnett=function(c,K,alpha)

{

cov=matrix(0.5,K,K)+diag(0.5,K)

int=1-pmvnorm(lower=rep(-Inf,K),upper=rep(c,K),mean=rep(0,K),sigma=cov)

return(as.double(int)-alpha)

}

#finddunnettcriticalvalues searches for the critical value that givens the correct fwer, and converts to p-values

finddunnettcriticalvalues=function(K,alpha)

{

criticalvalue=uniroot(fwer_dunnett,interval=c(0,qnorm(1-alpha/K)+0.01),K=K,alpha=alpha)

return(rep(1-pnorm(criticalvalue$root),K))

}

example:

> finddunnettcriticalvalues(4,0.05)

[1] 0.01537537 0.01537537 0.01537537 0.01537537

*Dunnett step-down procedure*

The Dunnett step-down procedure modifies the Dunnett procedure in a similar way that Holm’s procedure modifies Bonferroni.

With K null hypotheses, each with a p-value, Dunnett’s procedure works as follows:

1. order the p-values so that they go from lowest to highest;
2. compare the lowest p-value to the critical value found from Dunnett’s procedure with target FWER $\alpha$ and K arms, and reject the null hypothesis associated with that p-value if the p-value is below;
3. If the null hypothesis tested in step 2 was rejected, compare the second lowest p-value to the Dunnett critical value with target FWER $\alpha$ and K-1 arms , and reject the null hypothesis associated with that p-value if the p-value is below;
4. repeat step 3 for the third lowest, fourth lowest p-values etc, comparing the p-value of the jth lowest pvalue to the critical value from Dunnett’s procedure with target FWER $\alpha$ and number of arms $K-j+1$until a null hypothesis is not rejected.

R-code:

library(mvtnorm)

#fwer_dunnett returns the difference between the FWER for a given critical value and the target FWER

fwer_dunnett=function(c,K,alpha)

{

cov=matrix(0.5,K,K)+diag(0.5,K)

int=1-pmvnorm(lower=rep(-Inf,K),upper=rep(c,K),mean=rep(0,K),sigma=cov)

return(as.double(int)-alpha)

}

#finddunnettcriticalvalues searches for the critical value that givens the correct fwer, and converts to p-values

finddunnettcriticalvalues=function(K,alpha)

{

criticalvalue=uniroot(fwer_dunnett,interval=c(0,qnorm(1-alpha/K)+0.01),K=K,alpha=alpha)

return(rep(1-pnorm(criticalvalue$root),K))

}

finddunnettstepdowncriticalvalues=function(K,alpha)

{

criticalvalues=rep(0,K)

for(i in 1:(K-1)){criticalvalues[i]= finddunnettcriticalvalues(K-i+1,alpha)[1]}

criticalvalues[K]=alpha

return(criticalvalues)

}

Reference List

1. Bender R, Lange S: **Adjusting for multiple testing - when and how?** *Journal of clinical epidemiology* 2001, **54:** 343-349.

2. Dunnett CW: **A multiple comparison procedure for comparing several treatments with a control.** *Journal of the American Statistical Association* 1955, **50:** 1096-1121.

3. Dunnett CW, Tamhane AC: **Step-down multiple tests for comparing treatments with a control in unbalanced one-way layouts.** *Statist Med* 1991, **10:** 939-947.

4. Dunn OJ: **Multiple Comparisons among Means.** *Journal of the American Statistical Association* 1961, **56:** 52-64.

5. Holm S: **A simple sequentially rejective multiple test procedure.** *Scandinavian journal of statistics* 1979, 65-70.
